# The effect of a fermented soy beverage among patients with localized prostate cancer prior to radical prostatectomy

**DOI:** 10.1186/s12894-024-01483-y

**Published:** 2024-05-03

**Authors:** Soum D. Lokeshwar, Ather Ali, Theresa R. Weiss, Jesse Reynolds, Brian M. Shuch, Thomas Ferencz, Tassos C. Kyriakides, Wajahat Z. Mehal, Joseph Brito, Joseph Renzulli, Michael S. Leapman

**Affiliations:** 1 Department of Urology, Yale School of Medicine, 800 Howard Ave Fl 3, New Haven, CT 06519 USA; 2 Department of Pediatrics and Medicine, Yale School of Medicine, New Haven, CT USA; 3 Yale Center for Analytical Sciences, Yale School of Public Health, New Haven, CT USA; 4 Department of Urology, David Geffen School of Medicine at UCLA, Los Angeles, CA USA; 5 Yale Cancer Center, New Haven, CT USA; 6 Yale School of Medicine, New Haven, CT USA; 7 Department of Internal Medicine, Section of Digestive Diseases, Yale School of Medicine, New Haven, CT USA

**Keywords:** Prostate cancer, PSA, Fermented soy, Randomized controlled trial

## Abstract

**Background:**

Fermented soy products have shown to possess inhibitory effects on prostate cancer (PCa). We evaluated the effect of a fermented soy beverage (Q-Can®), containing medium-chain triglycerides, ketones and soy isoflavones, among men with localized PCa prior to radical prostatectomy.

**Methods:**

We conducted a placebo-controlled, double-blind randomized trial of Q-Can®. Stratified randomization (Cancer of the Prostate Risk Assessment (CAPRA) score at diagnosis) was used to assign patients to receive Q-Can® or placebo for 2–5 weeks before RP. Primary endpoint was change in serum PSA from baseline to end-of-study. We assessed changes in other clinical and pathologic endpoints. The primary ITT analysis compared PSA at end-of-study between randomization arms using repeated measures linear mixed model incorporating baseline CAPRA risk strata.

**Results:**

We randomized 19 patients, 16 were eligible for analysis of the primary outcome. Mean age at enrollment was 61, 9(56.2%) were classified as low and intermediate risk, and 7(43.8%) high CAPRA risk. Among patients who received Q-Can®, mean PSA at baseline and end-of-study was 8.98(standard deviation, SD 4.07) and 8.02ng/mL(SD 3.99) compared with 8.66(SD 2.71) to 9.53ng/mL(SD 3.03), respectively, (Difference baseline – end-of-study, *p* = 0.36). There were no significant differences in Gleason score, clinical stage, surgical margin status, or CAPRA score between treatment arms (*p* > 0.05), and no significant differences between treatment arms in end-of-study or change in lipids, testosterone and FACT-P scores (*p* > 0.05).

**Conclusions:**

Short exposure to Q-Can® among patients with localized PCa was not associated with changes in PSA levels, PCa characteristics including grade and stage or serum testosterone. Due to early termination from inability to recruit, study power, was not achieved.

## Introduction

Prostate cancer (PCa) is the most commonly diagnosed cancer in males and the second most common cause of cancer related deaths among men in the United States [1]. The burden from prostate cancer is substantial when appreciated on a global scale. Although often localized and slow-growing, treatments and disease monitoring are associated with significant toxicity, economic expenditure, and enduring risks of disease progression and cancer mortality. Given the protracted, yet potentially fatal, natural history of the disease, there is growing interest in low-risk interventions, particularly diet and lifestyle, in the tertiary prevention of prostate cancer. In particular, plant-based diets have been associated with numerous prostate cancer related outcomes including incidence, progression and mortality [2]. For example, in population-based studies, overall consumption of a plant based-diet was associated with lower risk of fatal prostate cancer, and in men < 65 was plant-based consumption was associated with lower risk of advanced, lethal, and fatal prostate cancer [3]. 

There is a large amount of data regarding the association of soy and cancer [4, 5]. The epidemiological data is mostly supportive with a reduced risk of breast cancer in Chinese populations with high dietary soy, and this has been confirmed in populations in the United States with relatively lower soy consumption [6, 7]. ZhenHua 851 or (Q-CAN® Plus or “QC”) is a fermented soy food product that contains medium chain triglycerides, ketones and soy isoflavones, and is available in commercial form. Uncontrolled studies and case reports support QC benefits in cancer progression and changes in activation markers in humans’ immune cells including changes in expression of CD3, CD4, and CD8 markers, as well increased NK cell activity [8, 9]. Specifically, these reports associate consumption of QC with reduced prostate specific antigen (PSA) velocity in men with PCa [10].

Patients express a strong interest in diet and lifestyle interventions in prostate cancer however there is a dearth of high-quality information available to inform clinical recommendations. Much of the available evidence addressing diet and lifestyle in prostate cancer has come in the form of anecdotal, or observational studies prone to methodological biases associated with unmeasured confounding and varied exposure. Therefore, the purpose of this study is to assess the efficacy of QC fermented soy on localized PCa prior to radical prostatectomy (RP) in a controlled trial setting.

## Methods

### Trial design and oversight

**Fig. 1:**
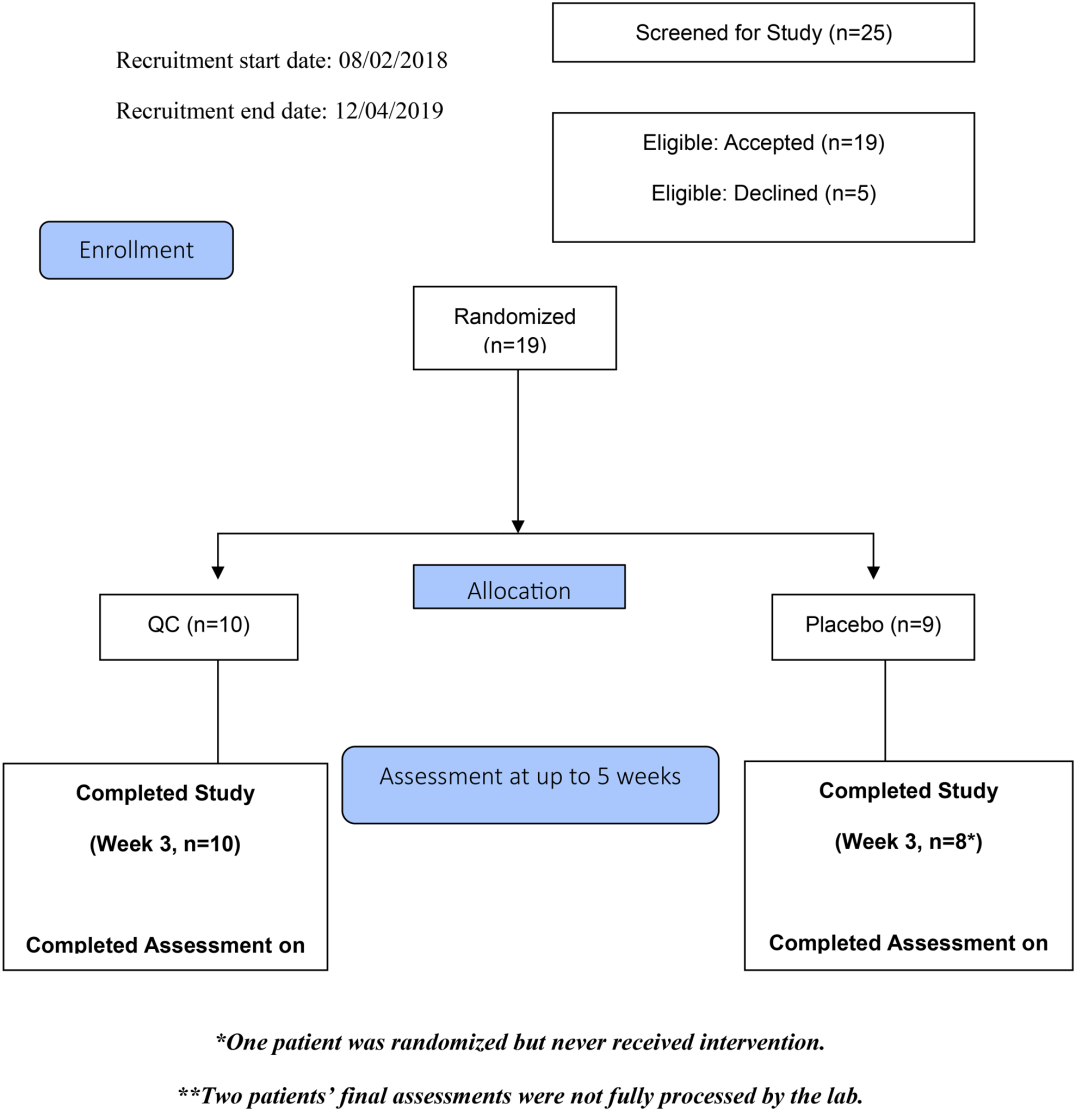
Study consort diagram

We performed a parallel group, double blind, randomized, clinical trial among patients with clinically localized prostate cancer who had elected initial treatment with RP (Fig. 1). The investigational product was Q-CAN® Plus (“QC”), a fermented soybean-derived phytochemical food supplement in liquid form [11]. In order to validate the Certificate of Analysis, the soy test agent (QC) underwent quarantine and independent third-party laboratory analysis (product characterization). QC is a commercially available beverage. The product’s primary constituents are the isoflavones genistin and daidzin (and respective metabolites of genistein and daidzein).

Study participants were randomized to one of two arms: to receive either Daily QC or placebo. Randomization was written and validated by the Yale Center for Clinical Investigation CTSA. Randomization was stratrified by risk level based on CAPRA score. After review of eligibility criteria, the blinded clinical trials manager accessed the trial management system to initate the randomization. Once a participant was deemed eligible, a blinded statistican performed the assignment to randomization and served as the liaison to the dispensing pharmacist. Patients in the intervention arm received Daily QC (QCAN fermented soy) as two 12.5 gram packets/day between the time of enrollment and the day prior to RP. The two packets were taken together once a day. This dose is calculated to contain 141 mg of genistin and 26 mg of genistein per mix of 8 oz. fluid. Patients in the control arm received daily matched isocaloric dose of brown-rice based placebo. These were taken at time of enrollment to RP (between 2 and 5 weeks prior to surgery). Participants were considered to have completed the study if they had completed all study visits. Study visits included an initial screening and baseline visit, a second visit for assignment of intervention, weekly phone calls thereafter to asses safety and adherence/dosing and a final assessment visit.

To be eligible for participation, we required that patients had histologically verified PCa at any stage, were ≥ 18 years of age and scheduled to be treated by RP within 2 to 5 weeks from screening and enrollment. Patients were excluded if they had previous (within 6 months of enrollment) or concurrent hormonal therapy or chemotherapy; specifically, treatment with 5-alpha reductase inhibitors (finasteride and dutasteride), history of hormone dependent malignancies, concomitant thyroid disease or currently taking thyroid hormone replacement medication, current high-dose soy consumption, micronutrient, or herbal supplements, on soy or vegetarian nutrition, or any other extreme dietary habits, currently taking oral anticoagulants or parenteral injection of low molecular weight heparin (enoxaparin), current or past history of any liver or pancreas disease, history of hypersensitivity to soy-containing products or malabsorption conditions.

The primary study endpoint was change in serum PSA defined as the difference between baseline PSA value and PSA value at the time of RP. Baseline was defined as the date that an eligible participant enrolled in the trial. The secondary endpoints were changes in other clinical and pathologic endpoints including prognostic Gleason Grade Group, stage, margin status, overall Cancer of the Prostate Risk Assessment (CAPRA) score, lipid levels, testosterone, and health related quality of life (FACT-P score). The target sample size per design was 72, with 36 patients in each arm. The assumptions used to derive the sample size using a repeated measures design was a PSA difference of 15 units, standard deviation of 20.3, Power of 0.8 and Type 1 error of 0.05. At the time of interim analysis, there were 19 participants randomized. One participant had not received intervention and for two participants there was no lab assessment made. The primary analysis was carried out using an ANOVA Repeated Measures Linear Mixed Model that modeled on treatment and time- this was done for main effect (treatment arm) and stratified (CAPRA risk score).

All patients provided written informed consent prior to administering any study procedures. Interim analysis and monitoring were performed by the principle investigators and presented and reviewed by the independent Data Safety Monitoring Board. This trial was approved by the Yale University institutional review board. CONSORT reporting guidelines were utilized [12].

### Statistical analysis

The intent-to-treat (ITT) efficacy analysis of endpoints included data from all randomized study participants. Safety was analyzed using data from all study participants who received at least one dose of QC. The analysis of safety included all events that occurred from the time of the first dose until the participant’s final assessment, just prior to RP. Final assessment of primary outcome, PSA as well as change of PSA utilized data from all participants who had end of study information available and complete laboratory analysis. A two-sided Type I error of 0.05 was used as the level of statistical significance.

The ITT analysis of the primary outcome was conducted using a repeated measures analysis or linear mixed model analysis using the stratification variable of baseline CAPRA risk. A secondary analysis the of primary outcome was performed using a generalized linear model adjusting for baseline PSA value. Continuous secondary outcomes, including lipids, free and total testosterone were analyzed using linear mixed models to assess mean changes (pre- to post-surgery). The Gleason score at RP and quality of life (FACT-P) after RP, both ordinal variables, were analyzed using a Wilcoxon Signed Rank test. Degree of tumor focality at RP was assessed with the chi-square method.

## Results

### Patients

From August 2, 2018 to December 4, 2019, a total of 25 patients were deemed eligible with 19 eligible patients electing to proceed and were randomized; 10 were assigned to the QC group and 9 were assigned to the placebo group. Baseline characteristics and demographics were well balanced between the two groups (Table 1). The median age of patients at time of enrollment in the QC cohort was 60.5 years and 60 years in the placebo cohort. There was no significant difference in the amount of time the product was taken between groups. At the time of final analysis, 16 patients were assessed for final measurement of primary outcome. Per CAPRA risk score, 9 (56.2%) patients were considered low and intermediate risk, and 7 (43.8%) were considered high CAPRA risk. Due to low enrollment, and after consultation and recommendation by the DSMB, an interim analysis was conducted after 19 participants had been enrolled, which suggested no statistically significant differences in the primary endpoint (Table 2). Therefore, the investigators and sponsors elected to terminate the study early.

**Table 1:** Baseline characteristics of included patients

Characteristic	Daily QC (*n* = 10)	Placebo (*n* = 9)	Total (*n* = 19)	Difference (95% CI)
**Age at Study**				
Mean (95% CL)	60.60 (55.66, 65.54)	62.89 (57.95, 67.83)	61.68 (58.50, 64.87)	2.28 (-8.77, 4.19)
Mean (SD)	60.60 (6.90)	62.89 (6.43)	61.68 (6.60)	
Median (IQR)	60.5 (58.0–66.0)	60.0 (58.0–69.0)	60.0 (58.0–69.0)	
**Education, number of years:**				
Mean (95% CL)	12.60 (11.42, 13.78)	14.11 (12.76, 15.47)	13.32 (12.44, 14.20)	1.51 (-3.16, 0.14)
Mean (SD)	12.60 (1.65)	14.11 (1.76)	13.32 (1.83)	
Median (IQR)	12.0 (12.0–14.0)	14.0 (12.0–16.0)	12.0 (12.0–15.0)	
Gender				
Male	10 (100.00%)	9 (100.00%)	19 (100.00%)	--
**Race self-identity**				
Black or African American	2 (20.00%)	2 (22.22%)	4 (21.05%)	− 2% (-45%, 49%)
White	8 (80.00%)	7 (77.78%)	15 (78.95%)	2% (-49%, 45%)
**Ethnicity**				
Hispanic or Latino	1 (10.00%)	0 (00.00%)	1 (5.26%)	10% (-39%, 19%)
Non-Hispanic	9 (90.00%)	9 (100.00%)	18 (94.74%)	-10% (-19%, 39%)
**Site**				
Lawrence & Memorial Hospital	5 (50.00%)	6 (66.67%)	11 (57.89%)	-16% (-37%, 70%)
Yale University	5 (50.00%)	3 (33.33%)	8 (42.11%)	17% (-70%, 37%)
Marital status:				
Married or Cohabiting	7 (70.00%)	8 (88.89%)	15 (78.95%)	-19% (-26%, 64%)
Not married/cohabitating	3 (30.00%)	1 (11.11%)	2 (10.53%)	19% (-64%, 26%)

**Table 2:** Analysis: repeated measures ANOVA using linear mixed models

	Treatment	
Time	QC	Placebo
***Least squares mean (standard error) of PSA***		
Baseline	8.97 (0.99)	8.83 (1.05)
End of Study	8.14 (1.01)	9.14 (1.10)

Type 3 Tests of Effects p values indicate the significance level of the main effects and interaction terms in the statistical model

Treatment: *p* = 0.76; Time: *p* = 0.61; CAPRA Risk: *p* = 0.04; Time*Treatment: *p* = 0.29

### Change in serum PSA

The difference in mean baseline PSA of the QC and Placebo groups was not statistically significant (8.98, 95% CI: 6.07–11.89 versus 8.66, 95% CI: 6.58–10.74, respectively). At the end of the study there was no statistically significant difference in PSA in the QC cohort compared to the placebo (8.02, 95% CI: 4.96–11.09 versus 9.53, 95% CI: 6.73–12.33) respectively). In terms of the difference between baseline and end of study PSA, there was no statistically significant difference between QC cohort and placebo (+ 0.85, 95% CI: -1.01–2.71 versus − 0.13, 95% CI: -1.46–1.19 respectively) (Table 3a). When stratified by CAPRA risk in the QC cohort compared to placebo, there was no statistically significant difference in baseline PSA, end of study PSA or PSA change from baseline to end of study (*p* > 0.05) (Table 3b).

**Table 3:** (A) Primary outcome bivariate analysis: PSA, (B) Primary outcome bivariate analysis: PSA, Stratified by CAPRA risk

	Treatment			
	QC	Placebo	Total	*P* Value
**(A)**				
**Baseline**				
N (N Missing)	10 (0)	9 (0)	19 (0)	
Mean (SD)	8.98 (4.07)	8.66 (2.71)	8.83 (3.40)	0.85
Mean (95% CL)	8.98 (6.07–11.89)	8.66 (6.58–10.74)	8.83 (7.19–10.47)	0.85
Median (Range)	9.3 (2.3–15.3)	8.2 (5.1–12.7)	8.7 (2.3–15.3)	0.9
**End of Study**				
N (N Missing)	9 [1]	7 [2]	16 [3]	
Mean (SD)	8.02 (3.99)	9.53 (3.03)	8.68 (3.57)	0.42
Mean (95% CL)	8.02 (4.96–11.09)	9.53 (6.73–12.33)	8.68 (6.78–10.59)	0.42
Median (Range)	8.0 (1.7–15.9)	10.8 (4.5–12.7)	8.5 (1.7–15.9)	0.34
**Difference (Baseline – End of Study**)				
N (N Missing)	9 [1]	7 [2]	16 [3]	
Mean (SD)	0.85 (2.42)	-0.13 (1.43)	0.42 (2.05)	0.36
Mean (95% CL)	0.85 (-1.01–2.71)	-0.13 (-1.46–1.19)	0.42 (-0.67–1.51)	0.36
Median (Range)	0.6 (-2.8–5.2)	-0.1 (-2.5–2.1)	0.4 (-2.8–5.2)	0.4
**(B)**				
***Low Risk***				
**Baseline**				
N (N Missing)	5 (0)	5 (0)	10 (0)	
Mean (SD)	6.26 (3.34)	8.08 (2.72)	7.17 (3.03)	0.37
Mean (95% CL)	6.26 (2.11–10.41)	8.08 (4.70–11.45)	7.17 (5.00–9.33)	0.37
Median (Range)	5.4 (2.3–10.6)	7.0 (5.8–12.7)	6.8 (2.3–12.7)	0.4
**End of Study**				
N (N Missing)	5 (0)	4 [1]	9 [1]	
Mean (SD)	6.50 (3.63)	8.07 (3.30)	7.20 (3.37)	0.53
Mean (95% CL)	6.50 (2.00–11.01)	8.07 (2.82–13.31)	7.20 (4.61–9.79)	0.53
Median (Range)	7.2 (1.7–10.1)	7.7 (4.5–12.4)	7.2 (1.7–12.4)	0.71
**Difference (Baseline – End of Study)**				
N (N Missing)	5 (0)	4 [1]	9 [1]	
Mean (SD)	-0.24 (1.70)	0.27 (1.38)	-0.02 (1.49)	0.64
Mean (95% CL)	-0.24 (-2.35–1.87)	0.27 (-1.93–2.47)	-0.02 (-1.16–1.13)	0.64
Median (Range)	0.5 (-2.8–1.5)	0.1 (-1.2–2.1)	0.3 (-2.8–2.1)	1
***High Risk***				
**Baseline**				
N (N Missing)	5 (0)	4 (0)	9 (0)	
Mean (SD)	11.69 (2.76)	9.40 (2.90)	10.67 (2.90)	0.26
Mean (95% CL)	11.69 (8.27–15.12)	9.40 (4.78–14.01)	10.67 (8.44–12.90)	0.26
Median (Range)	10.8 (8.6–15.3)	10.5 (5.1–11.5)	10.7 (5.1–15.3)	0.54
**End of Study**				
N (N Missing)	4 [1]	3 [1]	7 [2]	
Mean (SD)	9.92 (4.03)	11.49 (1.07)	10.59 (3.04)	0.55
Mean (95% CL)	9.92 (3.51–16.34)	11.49 (8.82–14.16)	10.59 (7.79–13.40)	0.55
Median (Range)	8.3 (7.2–15.9)	10.9 (10.8–12.7)	10.8 (7.2–15.9)	0.38
**Difference (Baseline – End of Study)**				
N (N Missing)	4 [1]	3 [1]	7 [2]	
Mean (SD)	2.21 (2.71)	-0.67 (1.59)	0.97 (2.62)	0.17
Mean (95% CL)	2.21 (-2.10–6.51)	-0.67 (-4.61–3.27)	0.97 (-1.45–3.40)	0.17
Median (Range)	2.1 (-0.6–5.2)	-0.1 (-2.5–0.6)	0.6 (-2.5–5.2)	0.22

### Secondary outcomes

There was no statistically significant difference between the groups in Gleason score at RP, clinical stage, surgical margins, percent of biopsy cores involved in cancer, extracapsular extension, lymph node invasion, seminal vesicle invasion or CAPRA score between the QC and placebo groups. In addition, there were no statistically significant differences between the QC arm and placebo arm in end-of-study or change in lipids, free and total testosterone (*p* > 0.05).

In the evaluation of quality-of-life measures, there was no significant difference in change of FACT-P self assessment survey score from baseline to end of study between the QC arm and the placebo arm (diff = 9.38, 95% CI: -1.22–19.98, *p* > 0.05). Furthermore, there was no significant difference in any component of the FACT-P including change of physical well-being (PWB), social well-being (SWB), emotional well-being (EWB), functional well-being (FWB), or Prostate cancer subscale, and sub scores.

### Safety

Adherence was assessed weekly and there was nearly 100% adherence (Table 4). A total of 4 adverse events in 3 patients in the QC arm and 1 adverse event in the placebo arm were reported in the study (Table 5). There was one serious adverse event (SAE) in the QC group requiring hospitalization: a participant presented with an enlarging aortic aneurysm and pneumonia during the trial period, but the SAE was deemed unrelated to QC administration. There were no serious adverse events that resulted in trial discontinuation in either cohort. There were no deaths during the trial period.

**Table 4:** Adherence and assessment of intervention over time

	QC n (%)	Placebo n (%)	Total n (%)
**Adherence to Intervention**			
< 50%	0	1 [11]	1 [5]
50-75%	1 [10]	0	1 [5]
> 75-90%	0	0	0
> 90%	9 (90)	8 (89)	17 (90)
**Adherence Assessment**			
< 50%	0	1 [11]	1 [5]
50-75%	1 [10]	0	1 [5]
75-90%	0	0	0
> 90%	9 (90)	8 (89)	17 (90)

*Note* Percent adherence is calculated using data from all Weeks of Follow-up (range 1–5) QC Adherence = (Observed Days of QC use/Expected Days of QC use)

**Table 5:** Adverse events

Event name	QC (n events, n patients)	Placebo (n events, n patients)	Total (n events, n patients)
Rash	[1]	(0, 0)	[1]
Aortic Aneurysm and Pneumonia*	[1]	(0, 0)	[1]
Nausea / Dizzyness	[1]	(0, 0)	[1]
Atrial Fibrillation	[1]	[1]	[2]
***Number of unique participants with AE***	[3, 4]	[1]	[4, 5]
AE led to study discontinuation	***0***	***0***	***0***

*Key* *denotes SAE

## Discussion

We performed a double blind, randomized, clinical trial to evaluate the effect of a soy product, QC, on PSA in patients with PCa prior to RP. We found no statistically significant difference in PSA changes in patients treated with QC compared to Placebo. We also found no difference in surgical specimens (including Gleason score, surgical margins, clinical stage, extracapsular extension, lymph node or semivesical invasion) or laboratory parameters (free, total and bioavailable testosterone, amylase, CRP, C-reactive protein, HDL, LDL, Lipase, ESR, TSH, triglycerides) in patients treated with QC. Furthermore, we found no changes in quality of life for patients treated with QC compared to placebo. There were no serious adverse events directly attributed to QC during the trial period. Although the trial had low recruitment, to our knowledge this is the first randomized control trial to evaluate a fermented soy product’s effect on PSA and surgical parameters in patients with PCa undergoing RP. Even though the study was terminated prematurely due to inability to recruit, and thus did not achieve the design power, contributes to a growing body of literature aimed toward evaluating low-toxicity interventions on PCa.

Soy isoflavones have been previously investigated for their relationship with PCa. In terms of risk of PCa, a systematic review and metanalysis summarized the results of 30 studies investigating soy product intake and PCa risk. The metanalysis included studies with patient self reported soy intake and studies which included soy products as an intervention. The metanalysis found a significant association of soy intake with reduced risk of PCa (Total soy food (*p* < 0.001), genistein (*p* = 0.008), daidzein (*p* = 0.018), and unfermented soy food (*p* < 0.001)) [13]. In patients with PCa, a recent systematic review assessed the role of genistein in PCa parameters, where the effect of genistein supplementation was investigated in two studies [14]. Lazarevic et al. investigated the effect of 30 mg of genistein daily for three to six weeks prior to RP compared to placebo in a randomized phase 2 clinical trial of 54 men with localized PCa. Compared to placebo, patients who were given genistein had statistically insignificant change in PSA (*p* = 0.051), however they had a significant difference in cellular response (*p* = 0.033), and cell proliferation (*p* < 0.001) [15]. However, this trial was evaluated and was found to have a high (completeness of outcome data) or unclear (allocation concealment, blinding of personell and outcome assessor) risk of bias on 4 out of 7 criteria but low risk of bias for sequence generation and blinding as well as selective outcome reporting. In contrast, in a relatively low risk of bias trial, randomization of 60 mg of genistein daily versus placebo for 12 weeks in men enrolled in watchful waiting, yielded no impact on mean change in PSA [16]. Similar to the findings of our clinical trial on fermented soy, Hamilton-Reeves et al. performed a double-blind, randomized, placebo-controlled trial to examine the effects of soy isoflavone capsules in patients with localized PCa for 6 weeks prior to RP. There was no statistically significant difference in change in PSA, testosterone, estrogen, or total cholesterol [17]. Unlike the in-vivo studies including our study and Hamilton-Reeves et al., Q-CAN has been shown to reduces viability and increase apoptosis of cancer cells in a fermentation, concentration and time dependent manner. This suggests that fermentation of soy results in the production of metabolites that can reduce cancer cell viability, and induce cellular apoptosis. Interestingly, it was shown, in-vitro, that these actions occurred independent of genistein content [18].

An important distinction of our study from the current available literature on PCa and soy, is that our trial investigated the fermented soy product QC specifically in a randomized control setting. Strengths of our study include the strict inclusion criteria and its randomized prospective nature. Additionally, unlike prior trials on soy and PCa, our study captured PSA results, laboratory values, surgical parameters, and quality of life measures.

There are several important limitations to note. Most significantly, this trial included a small sample size due to early terminiation and failed to achieve the protocol determined sample size. Therefore, adequate statistical power was not reached to assess the prespecified study endpoints. As additional considerations we did not account for longer-term outcomes or data regarding continued patient exposure to QC post-treatment. The trial also did not include feasibility measurements including patient acceptance and site demographics. Additionally, PSA is a surrogate measure which may not adequately represent cancer risk or response and may fail to identify a variety of antitumor effects. In addition, the length of the exposure was relatively short (a minimum of 2 weeks), which may also be inadequate to lead to measurable differences in clinical parameters. To account for these limitations, further trials should be cognizant of potential sample size limitations, considerations of exposure length, and should incorporate a wider range of clinical and biological endpoints. These findings of poor accrual highlight specific areas for feasibility assessment that should be conducted in future clinical trials of nutritional interventions in prostate cancer.

## Conclusion

A short exposure to a fermented soy beverage among patients with localized prostate cancer was not associated with changes in PSA levels or other prostate cancer characteristics, including grade and stage or serum testosterone. As a consequence of early termination, study power, per design, was not achieved.

## Data Availability

Data that support the findings of this study are available from the corresponding author upon reasonable request.
